# CD3zeta-mediated modulation of TCR signaling: a novel strategy for neuroprotection in retinal ganglion cell degeneration

**DOI:** 10.3389/fcell.2025.1652041

**Published:** 2025-09-03

**Authors:** Kexin Xu, Ning Yang, Lu Yu, Zhiyi Wang, Ningzhi Zhang, Wenye Cao, Yiqiao Xing

**Affiliations:** ^1^ Department of Ophthalmology, Renmin Hospital of Wuhan University, Wuhan, Hubei, China; ^2^ Department of Ophthalmology, Aier Eye Hospital of Wuhan University, Wuhan, Hubei, China

**Keywords:** glaucoma, retinal ganglion cell, T cell receptor, CD3ζ, optic nerve crush

## Abstract

**Purpose:**

Glaucoma, a leading cause of irreversible blindness, involves complex mechanisms beyond elevated intraocular pressure (IOP), including immune signaling dysregulation. This study focused on the role of the T-cell receptor (TCR) signaling pathway, particularly the CD3ζ chain, in retinal ganglion cell (RGC) degeneration and explored its potential as a neuroprotective target via immune modulation.

**Methods:**

A mouse optic nerve crush model was used to mimic glaucomatous neurodegeneration. CD3ζ knockdown was achieved using adeno-associated virus serotype 9 encoding short hairpin RNA. Retinal tissues were evaluated via immunofluorescence, Western blotting, and RT-qPCR to analyze the survival and death of RGCs and activation of key signaling pathways, including the MAPK and NF-κB pathways. Changes in inflammatory cytokine profiles were assessed to examine the broader impact of TCR modulation.

**Results:**

CD3ζ knockdown significantly improved RGC survival by reducing apoptosis and necroptosis. The neuroprotective effect of CD3ζ knockdown was accompanied by the restoration of MAPK signaling, specifically the phosphorylation of ERK and p38, and attenuation of NF-κB activation, indicated by decreased p65 phosphorylation. Furthermore, CD3ζ knockdown reduced the levels of proinflammatory mediators (IL-1β, TNF-α, and MMP-9) and increased that of the anti-inflammatory cytokine IL-10, creating a retinal microenvironment conducive to neuroprotection.

**Conclusion:**

This study demonstrates that CD3ζ plays a critical role in immune-mediated neurodegeneration in glaucoma. CD3ζ knockdown promotes RGC survival by modulating MAPK and NF-κB signaling pathways and regulating apoptosis and inflammation. These findings underscore the therapeutic potential of targeting TCR signaling to complement existing IOP-lowering treatments, offering a novel approach to preserving visual function in glaucoma.

## 1 Introduction

Glaucoma, a chronic neurodegenerative disease, is a leading cause of irreversible blindness worldwide. It is characterized by progressive loss of retinal ganglion cells (RGCs) and optic nerve damage (Jonas et al., 2017; Surgucheva et al., 2008). Elevated intraocular pressure (IOP) is the primary modifiable risk factor; however, lowering IOP does not frequently halt disease progression (Hakim et al., 2023). This suggests that additional mechanisms, including immune responses, oxidative stress, and vascular dysfunction, play significant roles in the pathogenesis of glaucoma (Mursch-Edlmayr et al., 2021; Shestopalov et al., 2021; Wang and Wei, 2023; Hoppe and Gregory-Ksander, 2024; Shi et al., 2024). Among the possible mechanisms, immune responses have been implicated as key contributors to RGC degeneration, positioning immune modulation as a potential therapeutic strategy (Yang et al., 2019). The immune system plays a double role in optic neurodegeneration, and thus, the regulation of immune responses has been the focus in the development of new neuroprotective strategies (Tezel, 2013).

In recent years, the effect of immune modulation on neurodegenerative changes in glaucoma via specific signaling pathways has received increasing attention. T cells, as critical components of the adaptive immune system, regulate a wide range of immune responses via the T-cell receptor (TCR) signaling pathway (Li et al., 1999; Alarcón et al., 2003; Chapman et al., 2020; Mariuzza et al., 2020). The TCR is a multi-subunit complex primarily composed of α and β chains, responsible for antigen recognition, and a CD3 complex, which transduces intracellular signals (Alarcón et al., 2003; Courtney et al., 2018; Gaud et al., 2018; Mariuzza et al., 2020). Among the CD3 subunits, the CD3ζ chain is important for signal propagation (Rudemiller et al., 2014; Ma et al., 2020; Dexiu et al., 2022). Upon antigen recognition, the immunoreceptor tyrosine-based activation motifs (ITAMs) in CD3ζ undergo phosphorylation, recruiting key signaling molecules, such as LCK and ZAP70.

The TCR-CD3 complex plays a pivotal role in adaptive immunity by initiating intracellular signaling cascades upon antigen recognition. CD3ζ, one of the essential ITAM-containing subunits, is indispensable for initiating and sustaining TCR signal transduction. Following TCR engagement, CD3ζ is rapidly phosphorylated by the Src family kinase LCK, which creates docking sites for ZAP70. Activated ZAP70 phosphorylates the linker for activation of T cells (LAT), thereby propagating signals to downstream cascades such as the MAPK and NF-κB pathways (Fischer et al., 2010; Thill et al., 2016; Lo et al., 2018; Damen et al., 2022; Fernández-Aguilar et al., 2023). These interactions activate downstream pathways, including the MAPK cascade, which regulates cell survival, proliferation, and immune responses (Borrie et al., 2017). The MAPK pathway, including ERK1/2 and p38 MAPK, plays a crucial role in neuronal survival and stress response. ERK1/2 is generally associated with cell survival, differentiation, and regeneration, whereas p38 is activated by stress stimuli and can induce apoptosis. Dysregulation of these pathways has been implicated in neurodegenerative diseases, including glaucoma.

The potential role of TCR signaling in neurodegeneration has recently garnered attention, with emerging studies linking aberrant immune activity to diseases such as glaucoma (Rossjohn et al., 2015; Shah et al., 2021). TCR signaling, specifically the CD3ζ chain, has recently been identified as a critical regulator of immune responses. Beyond its traditional role in T cells, CD3ζ is expressed in retinal neurons, including RGCs, where it influences axonal guidance, dendritic development, and synaptic plasticity (Benowitz et al., 2017). These findings indicate that dysregulated TCR signaling may exacerbate RGC degeneration in glaucoma, whereas its modulation could be neuroprotective (Rieck, 2013; Russo et al., 2016). To investigate the role of TCR signaling in glaucoma, we employed an optic nerve crush (ONC) model, a well-established experimental paradigm for studying RGC injury and neurodegeneration (Schwartz, 2004; Liu et al., 2021). ONC mimics key aspects of glaucomatous optic neuropathy, including RGC axon damage and subsequent neuronal loss, making it an ideal system for exploring therapeutic interventions. Using adeno-associated virus serotype 9 (AAV9)-mediated knockdown of CD3ζ, we evaluated its effects on RGC survival and elucidated the underlying mechanisms, focusing on the MAPK pathway and cell death processes, such as apoptosis and necroptosis.

This study highlights the vital roles of CD3ζ as a regulator of immune signaling and neuronal function in the retina. To the best of our knowledge, this is the first study to verify the specific mechanism through which CD3ζ affects neuroprotection in the TCR signaling pathway and to suggest its potential as an anti-inflammatory and neuroprotective target. By bridging the immunological and neurobiological aspects, our findings provide a foundation for developing targeted therapies that modulate TCR pathways to protect RGCs, offering a promising strategy for managing glaucoma beyond traditional IOP-lowering treatments.

## 2 Materials and methods

### 2.1 Animals

Male C57BL/6J mice, aged 7–8 weeks and weighing 18–20 g, were purchased from the Wuhan University Laboratory Animal Center. Before the experiments, the mice were acclimatized for 7 days in standard cages under a controlled environment with a 12-h light/dark cycle as well as constant temperature and humidity. Food and water were provided *ad libitum*.

All animal experiments were conducted in accordance with the ARVO Statement for the Use of Animals in Ophthalmic and Vision Research and were approved by the Institutional Animal Care and Use Committee (IACUC) of Wuhan University.

### 2.2 AAV9 plasmid construction and preparation

Recombinant AAV9 vectors were produced by General Biol (Anhui) Co. Ltd. The transfer plasmid encoded a short hairpin RNA (shRNA) targeting the CD3ζ gene driven by the U6 promoter and included a ZsGreen fluorescent reporter gene for visualizing transduction efficiency. The viral particles were packaged using a three-plasmid system with the AAV9 capsid and purified via iodixanol density gradient ultracentrifugation.

The purified AAV9 was suspended in sterile phosphate-buffered saline (PBS) and concentrated. The viral genome titer was determined using quantitative PCR (qPCR) targeting the U6 promoter sequence and was 3.5 × 10^12^ vector genomes/mL. The AAV solution was aliquoted and stored at −80 °C.

### 2.3 Intravitreal injection and ONC injury

Mice were anesthetized by intraperitoneal injection of 1% pentobarbital sodium (50 mg/kg). Two microliters of AAV suspension was injected into the vitreous body of the eye using micropipette-assisted intravitreal injection. ONC injury was performed 3 weeks after injection. The left optic nerve was exposed employing the temporal approach, and the nerve sheath was cut in the axial direction to separate the optic nerve to avoid damage to the central retinal artery. The optic nerve was held 0.5–1 mm behind the eyeball for 10 s. After surgery, erythromycin eye ointment was applied, and the mice were placed on an electric warming blanket until conscious. After awakening, they were fed normally in the vivarium. Mice were euthanized by intraperitoneal injection of 1% pentobarbital sodium. All surgical procedures were performed by the same trained investigator to ensure consistency.

### 2.4 Retinal histology and immunofluorescence

Mice were euthanized 7 days after ONC injury and 7 days after intravitreal AAV9 injection and ONC injury. They were infused with PBS to remove blood cells from the retina and with 4% paraformaldehyde for 15 min; the eyeballs were subsequently removed. Eyes were immersed in FAS eyeball fixative overnight, embedded in paraffin, and sliced into 10 μm-thick sections. For evaluating retinal thickness, sections were stained with hematoxylin and eosin (H&E) and imaged under a microscope. The thickness of the retinal plexiform layer (IPL) and retinal nerve fiber layer (RNFL) was measured at the same distance from the optic disk using ImageJ software. The ganglion cell layer (GCL) was counted in five consecutive zones in at least three different sections. For immunofluorescence, 10 μm cryosections were blocked with 5% goat serum and incubated overnight at 4 °C with primary antibodies (Supplementary Table S1). The sections were then incubated with Alexa Fluor-conjugated secondary antibodies (1:500) for 1 h at room temperature. Images were captured using a confocal microscope (Leica TCS SP8, Germany).

### 2.5 Western blot analysis

Retinal tissues were homogenized in RIPA lysis buffer containing protease and phosphatase inhibitors. The protein concentration was determined using the BCA assay. Equal amounts of protein (20 μg) were separated via SDS-PAGE on a 12.5% gradient gel and transferred onto polyvinylidene membranes prewetted with methanol. The membranes were blocked with rapid sealing fluid for 15 min at room temperature and incubated overnight at 4 °C with primary antibodies (Supplementary Table S1) against the target proteins. After washing the membranes with Tris-buffered saline with Tween-20 (TBST) three times, they were incubated with horseradish peroxidase-conjugated secondary antibodies (Servicebio, China, 1:5000) for 1 h at room temperature. Following another round of washing with TBST three times, bands were visualized using an enhanced chemiluminescence (Life-iLab, China) detection system. Bands were quantitated by analyzing the relative density of the exposed film using ImageJ software.

### 2.6 RGC labels and counts

The mice were euthanized after ONC injury, and RGC survival rate was determined. The retina was carefully separated from the fixed eyeball, dissected onto a flat retinal stent, and blocked with 5% goat serum in PBS containing 0.3% Triton X-100 at 4 °C for 24 h. The retina was then incubated with anti-Brn3a primary antibody (Synaptic Systems, Germany, 1:1000) at 4 °C for 48 h and subsequently with Alexa Fluor 594-labeled rabbit secondary antibody (Jackson Immuno Research Laboratories, United States of America, 1:500) for 24 h. Twelve regions in each retinal plane were imaged using a microscope, and immunopositive cells were counted using ImageJ software and averaged.

### 2.7 Quantitative reverse-transcription polymerase chain reaction

Total RNA was isolated from mouse retinas using the AFTSpin Animal tissue/cell Rapid RNA extraction kit (ABclonal), and the RNA concentration was determined. ABScript III RT premixed solution (RK20429, ABclonal) was used to reverse transcribe 1 μg RNA into cDNA. qPCR was performed in a 20 μL reaction mixture using Universal SYBR Green Fast qPCR mixture (RK21203, ABclonal) under the following conditions: 95 °C for 2 min, followed by 40 cycles of 95 °C for 15 s and 60 °C for 30 s. The primers used in the study are listed in Supplementary Table S2.

### 2.8 Statistical analyses

Statistical analyses were conducted using GraphPad Prism 9.0 (GraphPad Software, United States). Data normality was assessed using the Shapiro–Wilk test. Student’s t-test was used for two-group comparisons, whereas one-way ANOVA with Tukey’s *post hoc* test was applied for multiple group comparisons. Non-normally distributed data were analyzed using the Mann–Whitney *U* test or Kruskal–Wallis test with Dunn’s *post hoc* test. Bonferroni correction was applied for multiple comparisons wherever necessary. Results are presented as mean ± SEM, with *p* < 0.05 indicating a statistically significant difference.

## 3 Results

### 3.1 Reduction in RGCs and activation of CD3ζ following ONC

**FIGURE 1:**
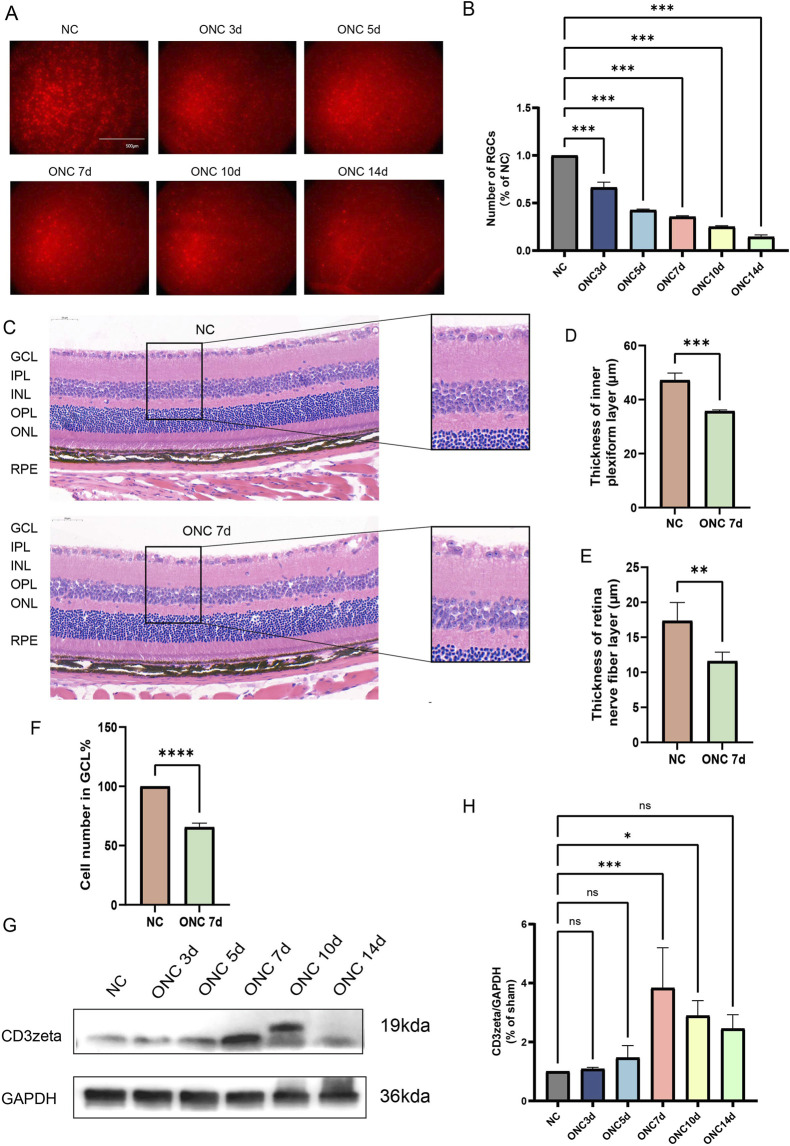
Dynamic changes in retinal ganglion cell (RGC) survival, retinal structure, and CD3ζ expression following optic nerve crush (ONC). **(A)** Representative immunofluorescence images of RGCs in flat-mounted retinas at different timepoints post-ONC injury (NC, ONC 3d, ONC 5d, ONC 7d, ONC 10d, and ONC 14d). RGCs were labeled with Brn3a (red). Scale bar, 500 μm. **(B)** Quantification of RGC counts normalized to the non-crush control (NC) group at indicated timepoints. Data are presented as mean ± SEM (*n* = 6 per group). ***p < 0.001, one-way ANOVA with Tukey’s *post hoc*test. **(C)** Representative hematoxylin and eosin-stained retinal sections from NC and ONC 7d groups showing structural changes in the ganglion cell layer (GCL), inner plexiform layer (IPL), inner nuclear layer, outer plexiform layer (OPL), outer nuclear layer (ONL), and retinal pigment epithelium layer. Scale bar, 50 μm. **(D,E)** Quantification of retinal IPL thickness **(D)** and retinal nerve fiber layer thickness **(E)** in NC and ONC 7d groups. Data are presented as mean ± SEM (*n* = 3 per group). ***p < 0.001, **p< 0.01, Student’s t-test. **(F)** Quantification of cell density in the GCL (% of NC group) in NC and ONC 7d groups. Data are presented as mean ± SEM (*n* = 3 per group). ****p < 0.0001, Student’s t-test. **(G)** Western blot analysis of CD3ζ expression in retinal tissues at various timepoints post-ONC (NC, ONC 3d, ONC 5d, ONC 7d, ONC 10d, and ONC 14d). Glyceraldehyde 3-phosphate dehydrogenase (GAPDH) was used as a loading control. **(H)** Quantification of CD3ζ expression normalized to GAPDH from Western blot analysis. Data are presented as mean ± SEM (*n* = 3 per group). *p < 0.05, ***p < 0.001; ns, not significant, one-way analysis of variance with Tukey’s *post hoc*test.

In the ONC model, partial or complete damage to the optic nerve, caused by application of clamp pressure to the optic nerve in animals, simulates pathological changes following central nervous system injury. This type of damage leads to the axons of RGCs being severed and serves as an effective model for simulating neurodegenerative changes in various human ophthalmic diseases such as glaucoma. Using Brn3a as a marker, we labeled RGCs in the mouse retina and quantified their survival at 3, 5, 7, 10, and 14 days post-ONC injury to capture the dynamic progression of RGC degeneration. Approximately 60% of RGCs were damaged by day 7 of ONC injury, and by day 14, only 10% remained viable (Figures 1A,B). H&E staining revealed thinning of the IPL and RNFL, along with a reduction in the number of cells in the GCL by day 7 of ONC injury (Figures 1C–F). Moreover, CD3ζ levels increased following ONC injury and peaked at day 7 post-injury, as evident from Western blotting (Figures 1G,H).

### 3.2 Expression of CD3ζ, LCK, and ZAP-70 in RGCs after ONC injury

To explore the activation of TCR following optic nerve injury, we focused on CD3ζ in the T-cell signaling pathway along with its downstream molecules, LCK and ZAP70.

**FIGURE 2:**
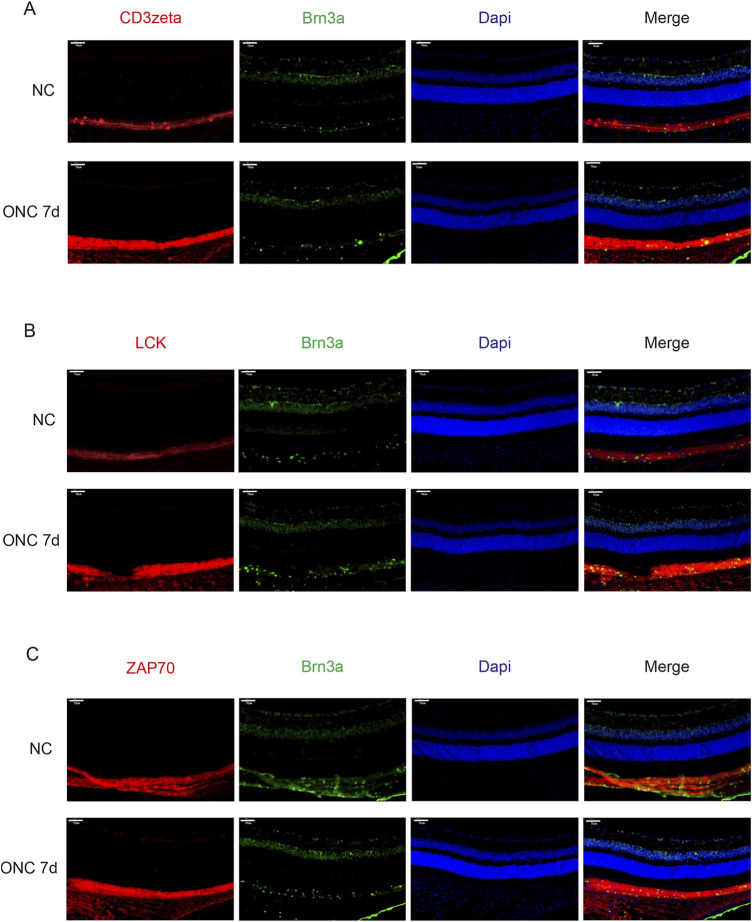
Expression of CD3ζ, LCK, and ZAP70 in retinal ganglion cells (RGCs) following optic nerve crush (ONC). **(A)** Representative immunofluorescence images showing CD3ζ (red) and Brn3a (green, RGC marker) in the ganglion cell layer (GCL) of the retina in normal control (NC) and ONC 7d groups. Merged images show colocalization of CD3ζ with RGCs in the GCL. Scale bar, 50 μm. **(B)** Representative immunofluorescence images showing LCK (red), Brn3a (green), and DAPI (blue) in the NC and ONC 7d groups. Increased LCK expression is observed in the ONC 7d group compared with that in the NC group. Scale bar, 50 μm. **(C)** Representative immunofluorescence images showing ZAP70 (red), Brn3a (green), and DAPI (blue) in the NC and ONC 7d groups. ZAP70 expression shows a similar increasing trend, with elevated signal intensity in the ONC 7 d group. Scale bar, 50 μm. Data are presented as mean ± SEM (*n* = 5 retinas per group).

First, we investigated whether CD3ζ is expressed in RGCs. Using immunofluorescence techniques, we observed colocalization of CD3ζ with the RGC marker Brn3a in the GCL, confirming that CD3ζ is expressed in RGCs (Figure 2A). Similar to the Western blotting results, we found that the expression of CD3ζ in the GCL increased following ONC injury.

Next, through immunofluorescence technology, we discovered that the downstream molecules LCK and ZAP70 were also expressed in GCL (Figures 2B,C). Moreover, their expression increased on the seventh day of ONC injury, similar to the trend of CD3ζ.

### 3.3 Knockdown of CD3ζ by AAV9-shRNA improves RGC survival after ONC injury

**FIGURE 3:**
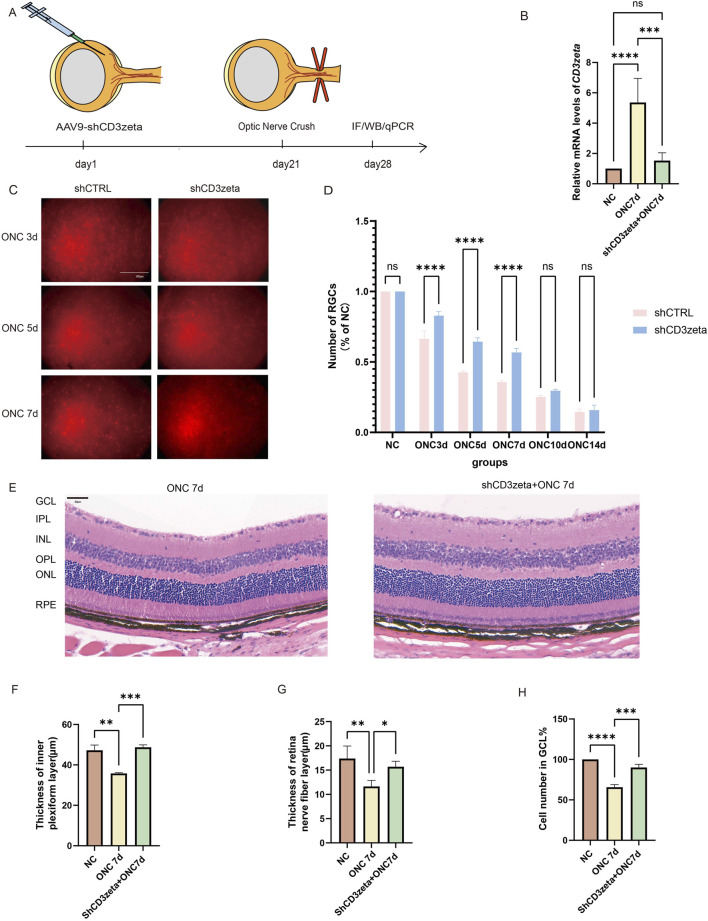
Effects of CD3ζ knockdown on retinal ganglion cell (RGC) survival and retinal structure following optic nerve crush (ONC). **(A)** Schematic of the experimental timeline, showing AAV9-shCD3ζ injection, ONC, and subsequent sample collection for immunofluorescence, Western blot, and quantitative PCR at various timepoints. **(B)** Quantification of relative CD3ζ mRNA expression in retinal tissues from NC and ONC 7d groups, with and without CD3ζ knockdown. Data are presented as mean ± SEM (*n* = 5). ***p < 0.001, ****p < 0.0001, one-way analysis of variance (ANOVA) with Tukey’s *post hoc* test. **(C)** Representative images of RGC labeling (Brn3a, red fluorescence) in the ONC 3d, ONC 5d, and ONC 7d groups, comparing the effects of CD3ζ knockdown (shCD3ζ) vs. control (shCTRL). Scale bar, 500 μm. **(D)** Quantification of RGC survival as a percentage of NC, comparing shCD3ζ and shCTRL groups across various ONC timepoints (*n* = 4). Data are presented as mean ± SEM. ****p < 0.0001, one-way ANOVA with Tukey’s *post hoc* test. **(E)** Representative hematoxylin and eosin staining images of retinal sections from NC and ONC 7d + shCD3ζ groups showing structural changes. Scale bar, 40 μm. **(F–H)** Quantification of changes in the retinal structure, including thickness of the inner plexiform layer **(F)**, retinal nerve fiber layer thickness **(G)**, and the number of cells in the ganglion cell layer **(H)** at 7 days post-ONC. Data are presented as mean ± SEM (*n* = 5). *p < 0.05, **p < 0.01, ***p < 0.001, ****p < 0.0001, one-way ANOVA with Tukey’s *post hoc* test. ns, not significant.

To investigate the role of CD3ζ in the survival of RGCs post-ONC, we specifically knocked down CD3ζ expression in RGCs using shCD3ζ, an AAV9-shRNA targeting CD3ζ. AAV9 carrying scrambled shRNA was used as a control (AAV9-shCtrl). We injected AAV9-shCD3ζ or AAV9-shCtrl into the vitreous body of 6-week-old C57BL/6 mice and performed ONC 3 weeks later. For analysis, the mice were euthanized 1 week after modeling (Figure 3A). AAV9 injection successfully reduced the mRNA levels of CD3ζ after ONC injury, as evident from the quantitative RT-PCR results (Figure 3B).

The RGC survival rates were evaluated using Brn3a as a marker. Immunofluorescence results showed that, compared to mice injected with shCtrl, those injected with shCD3ζ exhibited an increased number of RGCs at 3, 5, and 7 days after ONC injury, suggesting a protective effect of shCD3ζ on RGCs (Figures 3C,D).

Additionally, H&E staining of the retinas from AAV9-shCD3ζ-injected mice revealed that, compared to ONC-only mice without viral injection, the RNFL and IPL were thicker, and more cells were present in the GCL (Figures 3E–H).

These data indicate that CD3ζ knockdown significantly improved RGC survival and suggest a protective effect via regulation of key nodes in the TCR signaling pathway.

### 3.4 Suppression of CD3ζ reduces T-Cell signaling by modulating LCK and ZAP70 expression

**FIGURE 4:**
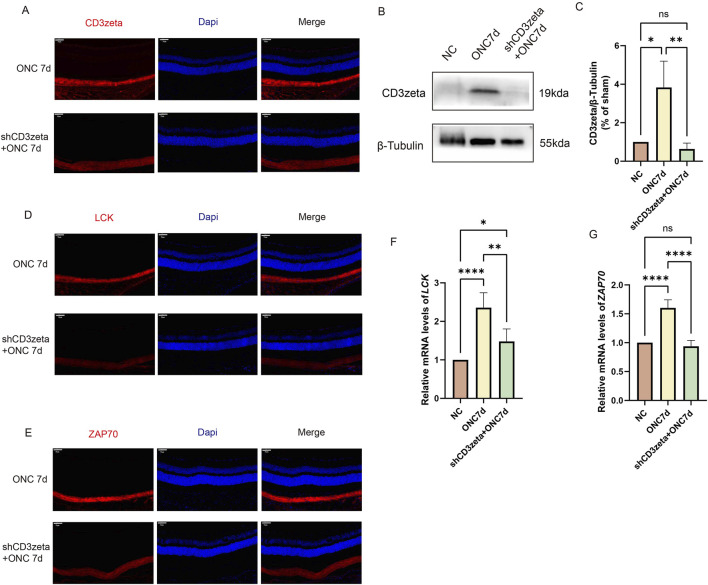
Effect of CD3ζ knockdown on the expression of CD3ζ, LCK, and ZAP70 in retinal ganglion cells following optic nerve crush (ONC). **(A)** Representative immunofluorescence images showing CD3ζ (red) and DAPI (blue, nuclear staining) in the ganglion cell layer (GCL) of retinal sections from ONC 7d and shCD3ζ + ONC 7d groups. CD3ζ expression is markedly reduced in the shCD3ζ + ONC 7d group. Scale bar, 50 μm. **(B)** Western blot analysis of CD3ζ expression in retinal tissues from NC, ONC 7d, and shCD3ζ + ONC 7d groups, with β-tubulin as the loading control. **(C)** Quantification of CD3ζ protein levels normalized to β-tubulin, showing significant reduction in the shCD3ζ + ONC 7d group compared to the ONC 7d group. Data are presented as mean ± SEM (*n* = 4). *p < 0.05, **p < 0.01, one-way analysis of variance (ANOVA) with Tukey’s *post hoc* test. **(D)** Representative immunofluorescence images of LCK (red) and DAPI (blue) in the GCL of ONC 7d and shCD3ζ + ONC 7d groups. Scale bar, 50 μm. **(E)** Representative immunofluorescence images of ZAP70 (red) and DAPI (blue) in the GCL of ONC 7d and shCD3ζ + ONC 7d groups. Scale bar, 50 μm. **(F)** Quantitative RT-PCR analysis of LCK mRNA levels in retinal tissues from NC, ONC 7d, and shCD3ζ + ONC 7d groups. LCK mRNA expression is significantly reduced in the shCD3ζ + ONC 7d group. Data are presented as mean ± SEM (*n* = 6). *p < 0.05, **p < 0.01, ****p < 0.0001, one-way ANOVA with Tukey’s *post hoc* test. **(G)** Quantitative RT-PCR analysis of ZAP70 mRNA levels in retinal tissues. ZAP70 expression follows a similar trend, with a significant reduction in the shCD3ζ + ONC 7d group. Data are presented as mean ± SEM (*n* = 6). ****p < 0.0001, one-way ANOVA with Tukey’s *post hoc* test. ns, not significant.

We examined the expression of CD3ζ following AAV9-shCD3ζ injection using immunofluorescence and Western blotting. The immunofluorescence intensity of CD3ζ was reduced compared with that in the non-injected group, confirming the suppression of CD3ζ expression (Figures 4A–C).

Next, we evaluated the expression of LCK and ZAP70 in the T-cell signaling pathway after suppressing CD3ζ. Immunofluorescence staining and quantitative RT-PCR results revealed that LCK and ZAP70 were upregulated following injury. However, their expression levels decreased upon inhibition of CD3ζ, indicating that suppression of CD3ζ expression inhibited the T-cell signaling pathway (Figures 4D–G).

### 3.5 Knockdown of CD3ζ restores MAPK pathway activity and suppresses NF-κB signaling after ONC injury

**FIGURE 5:**
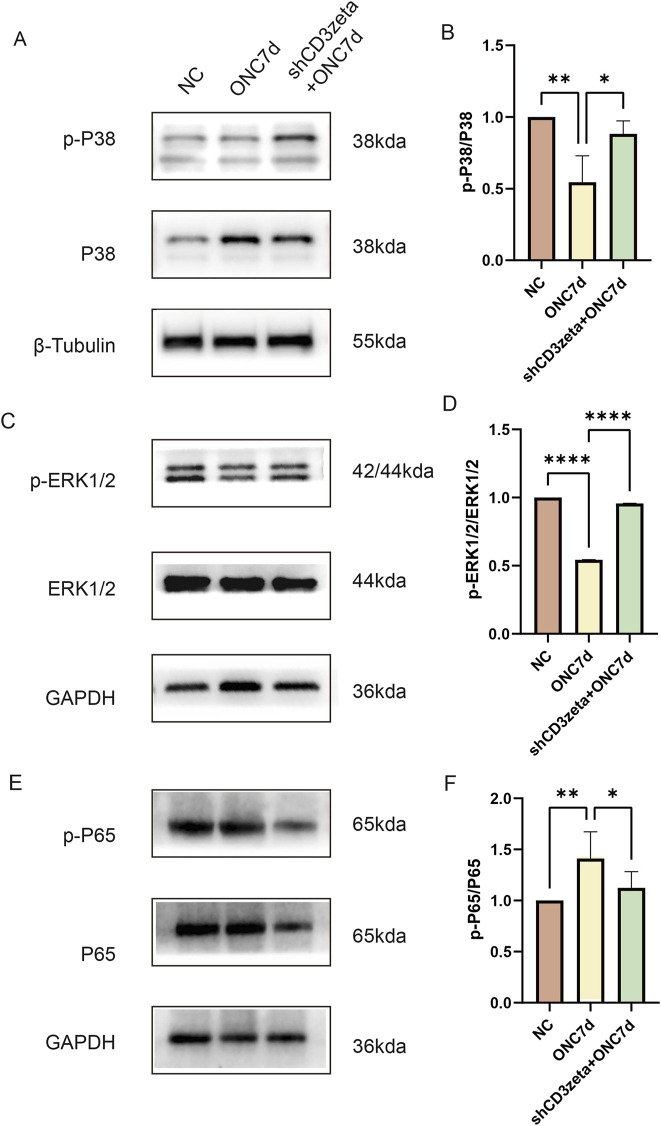
Effect of CD3ζ knockdown on the activation of MAPK and NF-κB pathways in retinal tissues following optic nerve crush (ONC). **(A)** Western blot analysis of phosphorylated p38 (p-p38) and total p38 expression in retinal tissues from NC, ONC 7d, and shCD3ζ + ONC 7d groups, with β-tubulin as the loading control. **(B)** Quantification of p-p38 levels normalized to total p38, showing significant restoration of p-p38 in the shCD3ζ + ONC 7d group. Data are presented as mean ± SEM (*n*= 5). *p < 0.05, **p < 0.01, one-way analysis of variance (ANOVA) with Tukey’s *post hoc*test. **(C)** Western blot analysis of phosphorylated ERK1/2 (p-ERK1/2) and total ERK1/2 expression across groups, with glyceraldehyde 3-phosphate dehydrogenase (GAPDH) as the loading control. **(D)** Quantification of p-ERK1/2 levels normalized to total ERK1/2, indicating significant recovery of ERK1/2 phosphorylation in the shCD3ζ + ONC 7d group compared to ONC 7d. Data are presented as mean ± SEM (*n* = 5). ****p < 0.0001, one-way ANOVA with Tukey’s *post hoc*test. **(E)** Western blot analysis of phosphorylated p65 (p-p65) and total p65 levels in NC, ONC 7d, and shCD3ζ + ONC 7d groups, with GAPDH as the loading control. **(F)** Quantification of p-p65 levels normalized to total p65, exhibiting reduced NF-κB pathway activation in the shCD3ζ + ONC 7d group. Data are presented as mean ± SEM (*n*= 5). *p < 0.05, **p < 0.01, one-way ANOVA with Tukey’s *post hoc*test.

To further explore the relationship between the TCR pathway and optic nerve protection, as well as its mechanism of action, we investigated the downstream pathways activated by TCR. After ONC injury, the phosphorylation levels of MAPK-related molecules, such as p38 and ERK, were downregulated. However, upon AAV9-shCD3ζ-mediated suppression of CD3ζ expression, the phosphorylation levels of p38 and ERK were restored to levels comparable to those in the control group (Figures 5A–D). This indicated that CD3ζ knockdown improved the MAPK pathway, which was inhibited after ONC injury. The restoration of MAPK pathway activity may be an important mechanism in realizing the neuroprotective effect after CD3ζ knockdown, which provides a clear direction for further research.

Previous studies have identified the activation of the CD3ζ chain as a critical step in initiating the NF-κB signaling pathway in T-cell activation. To further investigate this mechanism, we evaluated the phosphorylation levels of p65 as an indicator of NF-κB pathway activation. Silencing of CD3ζ significantly attenuated the elevated phosphorylation of p65 observed following ONC injury (Figures 5E,F).

### 3.6 Knockdown of CD3ζ can reduce cell death and cellular inflammation after ONC

**FIGURE 6:**
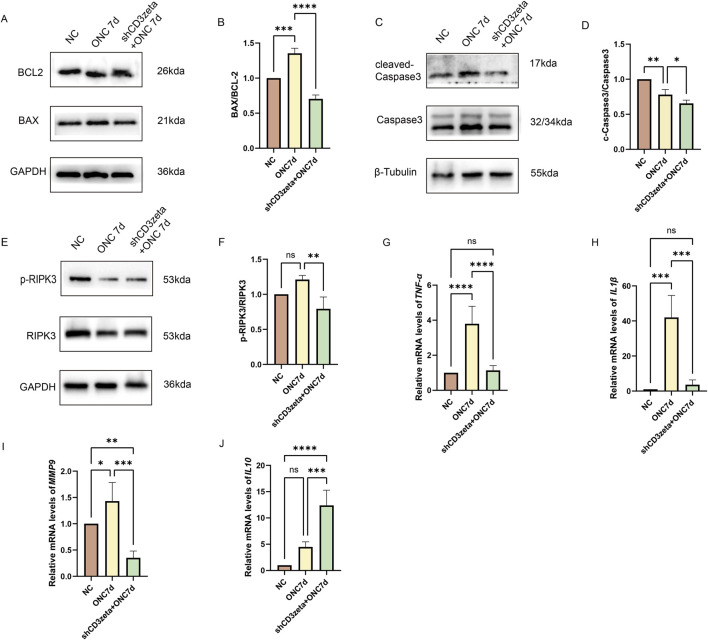
Effects of CD3ζ knockdown on apoptosis, necroptosis, and inflammatory mediator expression following optic nerve crush (ONC). **(A)** Western blot analysis of BCL-2 and BAX expression in retinal tissues from NC, ONC 7d, and shCD3ζ + ONC 7d groups, with glyceraldehyde 3-phosphate dehydrogenase (GAPDH) as the loading control. **(B)** Quantification of the BAX/BCL-2 ratio, indicating reduced proapoptotic signaling in the shCD3ζ + ONC 7d group compared to the ONC 7d group. Data are presented as mean ± SEM (*n* = 3). ***p < 0.001, ****p < 0.0001, one-way analysis of variance (ANOVA) with Tukey’s *post hoc*test. **(C)** Western blot analysis of cleaved-caspase3 and total caspase3 levels, with β-tubulin as the loading control. **(D)** Quantification of cleaved-caspase3/total caspase3 ratio, showing reduced apoptotic activity in the shCD3ζ + ONC 7d group. Data are presented as mean ± SEM (*n* = 3). *p < 0.05, **p < 0.01, one-way ANOVA with Tukey’s *post hoc*test. **(E)** Western blot analysis of phosphorylated RIPK3 (p-RIPK3) and total RIPK3 levels in retinal tissues, with GAPDH as the loading control. **(F)** Quantification of p-RIPK3/RIPK3 ratio, showing reduced necroptotic activity in the shCD3ζ + ONC 7d group. Data are presented as mean ± SEM (*n* = 3). **p < 0.01, one-way ANOVA with Tukey’s *post hoc*test. **(G,H)** Quantitative RT-PCR analysis of the mRNA levels of proinflammatory cytokines, including TNF-α **(G)** and IL-1β **(H)**, in NC, ONC 7d, and shCD3ζ + ONC 7d groups. CD3ζ knockdown significantly reduced the expression of TNF-α and IL-1β compared with that in ONC 7d. Data are presented as mean ± SEM (*n* = 6). ***p < 0.001, ****p < 0.0001, one-way ANOVA with Tukey’s *post hoc*test. **(I,J)** Quantitative RT-PCR analysis of MMP9 **(I)** and IL-10 **(J)** mRNA levels. While MMP9 expression was reduced, IL-10, an anti-inflammatory cytokine, was significantly upregulated in the shCD3ζ + ONC 7d group compared with that in the ONC 7d group. Data are presented as mean ± SEM (*n* = 6). *p < 0.05, **p < 0.01, ***p < 0.001, ****p < 0.0001, one-way ANOVA with Tukey’s *post hoc*test. ns, not significant.

ONC-induced RGC loss is closely linked to intrinsic and extrinsic apoptotic pathways. At 7 days post-ONC, the increased BAX/BCL-2 ratio indicated upregulation of proapoptotic proteins and downregulation of antiapoptotic proteins, whereas the increased cleaved-caspase3/caspase3 ratio confirmed the activation of apoptosis. AAV9-shCD3ζ injection significantly reduced both ratios, indicating that CD3ζ knockdown partially inhibited apoptosis by mitigating mitochondrial outer membrane permeabilization and downstream caspase activation (Figures 6A–D). Notably, the cleaved-caspase3/caspase3 ratio in the shCD3ζ group was lower than that in the control, possibly reflecting a decrease in basal apoptotic signaling due to CD3ζ-associated immunomodulation.

In addition to apoptosis, ONC-induced retinal damage also triggers necroptosis, characterized by RIPK3 phosphorylation. Although RIPK3 phosphorylation did not increase significantly at 7 days post-ONC, CD3ζ knockdown led to a marked reduction in the levels of phosphorylated RIPK3, indicating suppression of necroptotic pathways (Figures 6E,F).

Furthermore, CD3ζ knockdown significantly altered the retinal immune microenvironment. Proinflammatory factors (TNFα, IL-1β, and MMP9) were downregulated, whereas anti-inflammatory IL-10 levels were upregulated, as confirmed through the quantitative RT-PCR analysis. These changes indicate that CD3ζ knockdown attenuated neurodegeneration by modulating inflammatory responses and restoring immune homeostasis in the retina (Figures 6G–J).

## 4 Discussion

Glaucoma is a complex neurodegenerative disease characterized by progressive optic nerve damage and visual field loss, making it one of the leading causes of irreversible blindness worldwide. Although elevated IOP remains the primary modifiable risk factor, its treatment is insufficient in fully halting disease progression. This highlights the importance of exploring additional mechanisms, particularly immune responses, in the pathogenesis of glaucoma (Tezel, 2013; He et al., 2019). Recent studies revealed the neuroprotective potential of immune regulation in glaucoma (Adornetto et al., 2019; Au and Ma, 2022). Our study further supports this hypothesis by demonstrating the potential role of TCR signaling in the survival of RGCs.

Notably, we observed a significant reduction in the mRNA levels of LCK and ZAP70, two critical kinases in the TCR signaling pathway. This reduction likely reflects feedback inhibition or transcriptional regulation in the TCR signaling cascade. A possible explanation is that CD3ζ knockdown disrupts the phosphorylation of its ITAM motifs, leading to decreased downstream signaling and reduced transcriptional activity of LCK and ZAP70. This attenuation of TCR signaling could create a feedback loop to conserve cellular resources when signaling demand is reduced. Additionally, epigenetic modifications may play a role, as CD3ζ suppression affects chromatin accessibility and histone acetylation at the promoter regions of these genes, leading to reduced expression. Future studies using chromatin immunoprecipitation or RNA-seq techniques will be valuable in elucidating the specific transcriptional changes associated with CD3ζ modulation (Lysechko and Ostergaard, 2005; Methi et al., 2008; Damen et al., 2022).

We observed that CD3ζ knockdown resulted in the restoration of MAPK signaling, which plays a crucial role in cell survival and regeneration. The activation of ERK and p38 MAPK pathways following CD3ζ suppression was associated with enhanced neuronal survival and axonal repair (Kikuchi et al., 2000). This dual activation suggests a synergistic effect, wherein ERK promotes neuronal survival and p38 mitigates the inflammatory damage often seen in neurodegenerative diseases. These results are consistent with previous reports demonstrating that modulation of the MAPK pathway promotes axon regeneration and neuroprotection in various injury models, including optic nerve injury (Baeza-Raja and Muñoz-Cánoves, 2004; Galindo-Romero et al., 2021). Therefore, our findings support the notion that MAPK signaling is a central pathway through which CD3ζ exerts its neuroprotective effects. Although ERK and p38 were examined in this study, JNK—another member of the MAPK family—was not investigated. Since JNK is known to mediate neuronal apoptosis and inflammation, future studies will assess whether CD3ζ also regulates JNK activation in the context of optic nerve injury.

In addition to MAPK, we observed a reduction in NF-κB activation, as evidenced by decreased p65 phosphorylation. Activation of NF-κB, a key regulator of inflammation, is typically associated with the expression of proinflammatory cytokines, such as TNF-α and IL-1β, which exacerbate neuronal damage in glaucoma. By inhibiting NF-κB activation, CD3ζ knockdown not only attenuated the inflammatory response but also promoted a more favorable environment for the survival of RGCs. This finding is particularly significant, as it provides a mechanistic link between immune modulation and neuroprotection in glaucoma (Nakazawa et al., 2002).

The expression patterns of BAX/BCL-2 and phosphorylated RIPK3 (p-RIPK3) further support the involvement of both apoptotic and necroptotic pathways in ONC-induced RGC degeneration. The observed increase in the pro-apoptotic marker BAX and the concurrent decrease in anti-apoptotic BCL-2 in the ONC group indicate a shift toward mitochondrial-mediated apoptosis. CD3ζ knockdown markedly attenuated this imbalance, suggesting that silencing CD3ζ helps preserve mitochondrial integrity and promotes RGC survival. Similarly, the elevation of p-RIPK3/RIPK3 ratio in ONC retinas reflects necroptotic activation. The reversal of this increase in the CD3ζ knockdown group implies that CD3ζ signaling may also modulate necroptosis, potentially through upstream regulation of innate immune signaling and inflammatory cascades. These findings reinforce the hypothesis that CD3ζ influences RGC fate by modulating both apoptotic and necroptotic cell death pathways. Interestingly, cleaved caspase-3 expression was markedly reduced in the CD3ζ-knockdown group and even lower than that in uninjured controls. This may reflect a basal suppression of apoptotic tone caused by CD3ζ deficiency, potentially through dampening of inflammatory signaling or microglial activation.

We also examined the effect of CD3ζ knockdown on inflammatory mediator levels. We found a significant reduction in proinflammatory mediators, IL-1β, TNF-α, and MMP-9, and a concurrent increase in the anti-inflammatory cytokine IL-10. This shift in the cytokine profile indicates that CD3ζ not only modulates immune signaling but also plays a role in rebalancing the immune response to promote neuroprotection. The upregulation of IL-10 is particularly noteworthy, as it inhibits the inflammatory cascades driven by TNF-α and IL-1β and promotes tissue repair (Lawrence, 2009; Yu et al., 2020; Zhang et al., 2023).

From a translational perspective, the therapeutic targeting of CD3ζ signaling raises both promise and challenges. Given that CD3ζ is a central component of the TCR complex, its systemic modulation could potentially interfere with adaptive immune responses, leading to unintended immunosuppression or immune dysregulation. Therefore, a key consideration for clinical application is the development of tissue-specific delivery strategies, such as retina-targeted AAV vectors or RGC-selective promoters, to restrict modulation effects to ocular tissues while minimizing systemic exposure. Moreover, the long-term effects of CD3ζ modulation on retinal immune homeostasis and microglial activity remain to be elucidated and will be an important focus in future translational studies. These efforts will be essential for assessing the feasibility of CD3ζ-based interventions in chronic glaucoma patients, particularly those with concurrent systemic immune disorders or those undergoing immunotherapy.

It is also important to note that the ONC model used in this study represents an acute injury and does not fully replicate the chronic, progressive elevation of intraocular pressure that defines the clinical course of glaucoma. While the ONC model effectively mimics key features of RGC axon damage and neuronal loss, it lacks the sustained immune activation and pressure-related stress observed in chronic disease. Future studies will validate the neuroprotective effects of CD3ζ modulation in chronic IOP elevation models, such as the microbead-induced ocular hypertension model, to better assess translational relevance.

In conclusion, our study demonstrates that CD3ζ plays a pivotal role in modulating immune signaling pathways that contribute to RGC degeneration and neuroprotection in glaucoma (Galindo-Romero et al., 2021; Shi et al., 2024). By targeting CD3ζ and modulating both MAPK and NF-κB pathways, we could enhance the survival of RGCs and reduce inflammation, providing a promising therapeutic strategy for managing glaucoma. Future studies should explore the long-term effects of CD3ζ modulation and further investigate its potential as an adjunctive therapy alongside traditional IOP-lowering treatments to preserve visual function in patients with glaucoma (Salkar et al., 2024).

## Data Availability

The original contributions presented in the study are included in the article/Supplementary Material, further inquiries can be directed to the corresponding authors.
