# Supplementary material for: CD3zeta-mediated modulation of TCR signaling: a novel strategy for neuroprotection in retinal ganglion cell degeneration
Source: Front Cell Dev Biol. 2025 Sep 3;13:1652041. doi: 10.3389/fcell.2025.1652041 (PMC12440959; doi:10.3389/fcell.2025.1652041)
Supplement: Supplementary file 1 [file Table1.docx]

Supplementary Material

**
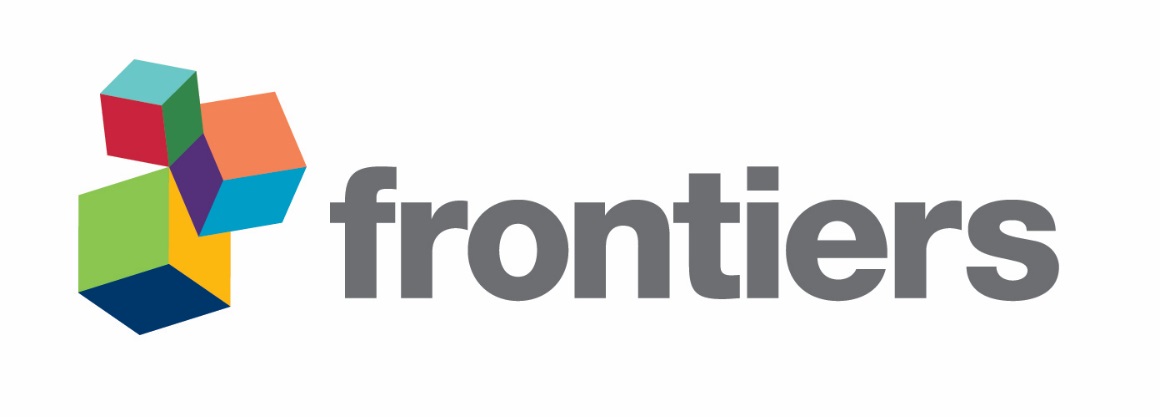
**

**Supplementary Table 1.** Primary antibodies used in western blotting and immunofluorescence applications

| Primary antibodies | Manufacturer | Application | Dilution ratio |
| --- | --- | --- | --- |
| GAPDH | HUABIO | WB | 1:3000 |
| β-Tubulin | Abmart | WB | 1:3000 |
| CD3ζ | Thermo | WB/IF | 1:2000/1:200 |
| ZAP70 | CST | IF | 1:200 |
| LCK | CST | IF | 1:200 |
| p-P38 | CST | WB | 1:1000 |
| P38 | CST | WB | 1:1000 |
| p-ERK1/2 | Abmart | WB | 1:2000 |
| ERK1/2 | Abmart | WB | 1:2000 |
| p-P65 | CST | WB | 1:1000 |
| P65 | CST | WB | 1:1000 |
| BCL2 | HUABIO | WB | 1:2000 |
| BAX | HUABIO | WB | 1:5000 |
| c-Caspase3 | CST | WB | 1:1000 |
| Caspase3 | CST | WB | 1:1000 |
| p-RIPK3 | Abclonal | WB | 1:1000 |
| RIPK3 | Novus | WB | 1:1000 |

CST, Cell Signaling Technology; WB, western blotting; IF, immunofluorescence

**Supplementary Table 2.** Primer sequences used in PCR analysis

| Primer name | Sequence (5′–3′） |
| --- | --- |
| Mouse CD247-F | GTGAGATCGGCACAAAAGGC |
| Mouse CD247-R | CAGTGCTGAGACCCTGGTAAA |
| Mouse LCK-F | CAAAGTTGAGCCGTCCTTGC |
| Mouse LCK-R | CCGTTGTAGTACCCCATCCAC |
| Mouse Zap70-F | AAGAGGATGGAATGTCCGCC |
| Mouse Zap70-R | GGGCGATCCTCCCACTTGTA |
| Mouse IL-1β-F | ATGCCACCTTTTGACAGTGAT |
| Mouse IL-1β-R | AAGGTCCACGGGAAAGACAC |
| Mouse IL-6-F | AGAGACTTCCATCCAGTTGCC |
| Mouse IL-6-R | CCGGACTTGTGAAGTAGGGAA |
| Mouse Mmp9-F | CGACTTTTGTGGTCTTCCCC |
| Mouse Mmp9-R | TAGCGGTACAAGTATGCCTCTG |
| Mouse IL10-F | GCTCTTACTGACTGGCATGAG |
| Mouse IL10-R | CGCAGCTCTAGGAGCATGTG |
| Mouse GAPDH-F | TCGCTCCTGGAAGATGGTGAT |
| Mouse GAPDH-R | CAGTGGCAAAGTGGAGATTGTTG |

**Supplementary Figure 1.** AAV9 vector map and sequence validation of CD3ζ shRNA construct. (A) Schematic representation of the recombinant adeno-associated virus serotype 9 (AAV9) vector used for CD3ζ knockdown. The vector (VA009-AAV-U6-MCS-CMV-EGFP) includes key regulatory elements such as the AAV2 inverted terminal repeats (ITRs), U6 promoter driving the short hairpin RNA (shRNA) cassette targeting CD3ζ, cytomegalovirus (CMV) promoter for EGFP reporter expression, and WPRE and hGH poly(A) signal to enhance transgene expression.

(B) Sequencing result of the shRNA expression cassette. The highlighted red box indicates the inserted target sequence specific to CD3ζ, confirming successful incorporation into the U6 promoter-driven backbone. This sequence corresponds to the validated shRNA sequence used in our study to achieve effective gene silencing in retinal ganglion cells.
