# Supplementary figures and images for: CD3zeta-mediated modulation of TCR signaling: a novel strategy for neuroprotection in retinal ganglion cell degeneration
Source: Front Cell Dev Biol. 2025 Sep 3;13:1652041. doi: 10.3389/fcell.2025.1652041 (PMC12440959; doi:10.3389/fcell.2025.1652041)

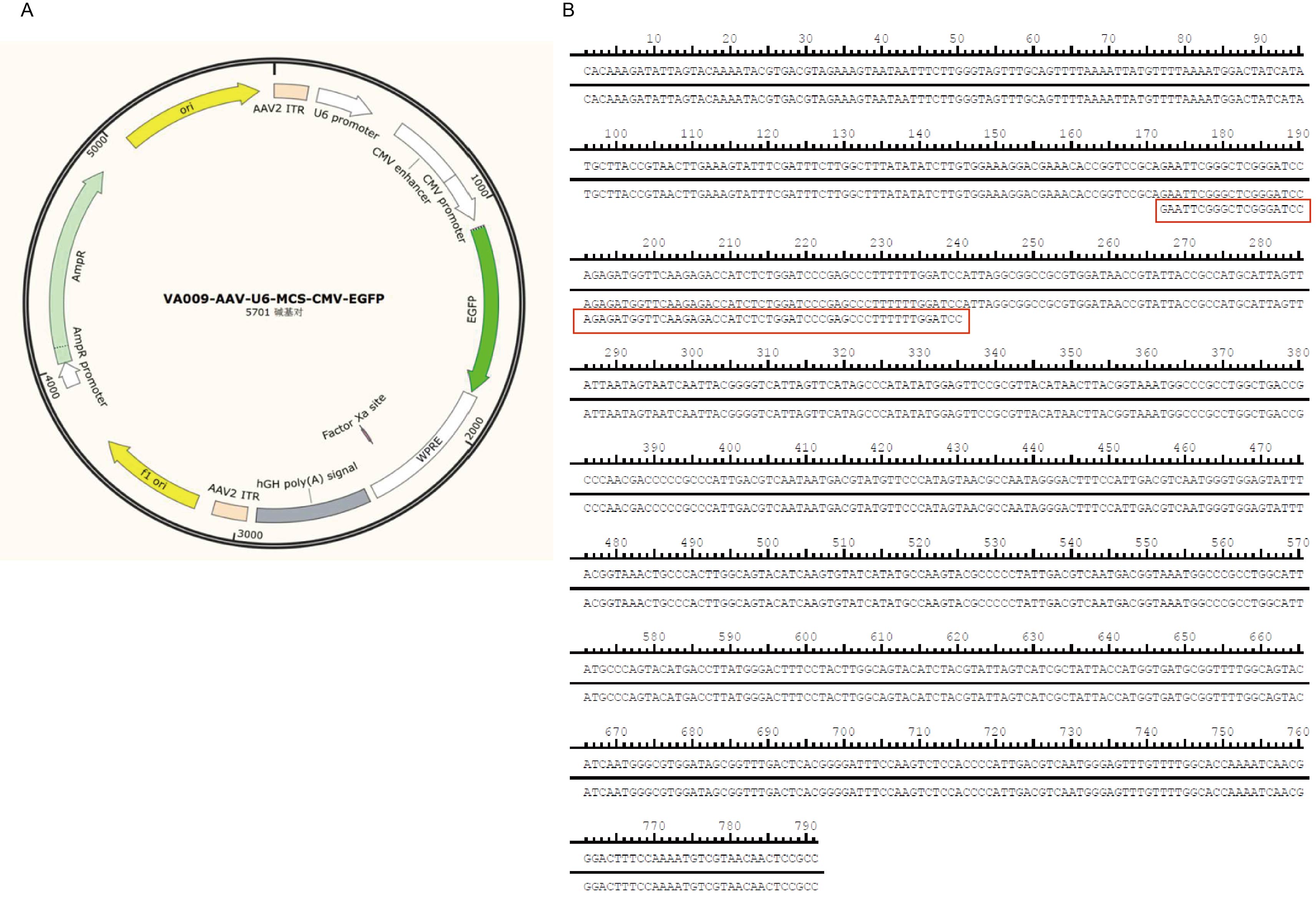

Supplement: Supplementary file 2 [file Image1.jpeg]
